# Artifacts in Optical Coherence Tomography Angiography

**DOI:** 10.18502/jovr.v16i2.9091

**Published:** 2021-04-29

**Authors:** Pasha Anvari, Maryam Ashrafkhorasani, Abbas Habibi, Khalil Ghasemi Falavarjani

**Affiliations:** ^1^Eye Research Center, The Five Senses Institute, Rassoul Akram Hospital, Iran University of Medical Sciences, Tehran, Iran; ^2^Stem Cell and Regenerative Medicine Research Center, Iran University of Medical Sciences, Tehran, Iran

**Keywords:** Artifact, Artefact, Capillary Plexus, Image Quality, Optical Coherence Tomography Angiography, Projection, Segmentation, Vessel Density

## Abstract

We performed a comprehensive search of the published literature in PubMed and Google Scholar to identify types, prevalence, etiology, clinical impact, and current methods for correction of various artifacts in optical coherence tomography angiography (OCTA) images. We found that the prevalence of OCTA image artifacts is fairly high. Artifacts associated with eye motion, misidentification of retinal layers, projections, and low optical coherence tomography signal are the most prevalent types. Artifacts in OCTA images are the major limitations of this diagnostic modality in clinical practice and identification of these artifacts and measures to mitigate them are essential for correct diagnosis and follow-up of patients.

##  INTRODUCTION

Optical coherence tomography angiography (OCTA) is an imaging method that provides three-dimensional images from the microcirculation of the retina, choroid, and optic nerve head. Considering its noninvasive nature and fast acquisition time, OCTA has gained priority over fluorescein angiography (FA), the traditional standard technique for evaluating retinal vasculature, for several retinal and choroidal disorders.^[1,2,3,4,5,6]^ In addition, depth-resolved OCTA images have improved our understanding of the pathogenesis, classification, and management of posterior segment diseases.^[7,8]^

Despite prominent advantages of OCTA, different types of artifacts may limit the interpretation and clinical application of this imaging modality.^[9]^ Previous studies have reported several types of artifacts impacting OCTA-derived metrics.^[9,10,11,12,13,14]^ Overall, the prevalence of artifacts ranges from 72 to 100%.^[13,14,15,16,17]^ Although various artifacts in OCTA images (e.g., segmentation artifact, shadow artifact, etc.) are similar to those reported in OCT images, several types of the artifacts are unique to OCTA. Recognition and minimizing or controlling such artifacts is crucial to avoid clinical misinterpretation^[18]^. This study aimed to review the literature describing OCTA artifacts.

##  METHODS

A comprehensive literature search was performed on August 9, 2020, in PubMed and Google Scholar using the key words “OCT Angiography" or “Optical Coherence Tomography Angiography" or “OCTA" and “Artifact" or “Artifacts" or “artefact" or “artefacts" to extract English-language original and review articles. Two researchers evaluated the abstracts and included relevant articles. Case reports were excluded.

##  RESULTS

Of the 9,220 and 206 studies found in Google Scholar and PubMed, respectively, 59 studies were found eligible and included for this review.

### Prevalence 

The prevalence of artifacts varied based on the OCTA device, setting, type of artifacts studied, and underlying disease [Table 1]. Ghasemi Falavarjani et al^[13]^ assessed OCTA images of 57 eyes including healthy subjects, individuals with age-related macular degeneration (AMD), and cystoid macular edema secondary to diabetic retinopathy (DR) or retinal vein occlusion (RVO). In 89.4% of images, at least one artifact was found. The most prevalent error was banding artifact (89.4%) followed by segmentation (61.4%), motion (49.1%), unmasking (15.8%), blink (8.8%), vessel doubling (1.7%), masking (1.7%), and out-of-window artifacts (1.7%). In diseased eyes, banding, motion, and segmentation artifacts were more prevalent. Chen and colleagues^[19]^ reviewed 60 OCTA images for motion artifacts as horizontal dark lines or bands not visible on OCT reflectivity maps. These lines were evident in 100% of the OCTA images from the outer retina, 90% of images from Sattler's layer, and 70% of the images from Haller's layer.

**Table 1:** The prevalence of optical coherence tomography angiography artifacts


**Authors**	**Design**	**Studied group**	**Artifacts reported**	**Device type and Software version**
Chen et al, 2016^[19]^	Prospective observational study	Normal Retinal diseases Glaucoma	Motion (100%) Fringe washout (100%) Projection (100%) Masking and unmasking (100%) Stromal decorrelation signal (100%) Retinal vessels duplication (5%)	RTVue XR Avanti system (Optovue Inc., CA, US)
Ghasemi Falavarjani et al, 2016^[13]^	Retrospective observational study	Age-related macular degeneration Diabetic retinopathy Retinal vascular occlusions	Band (89.4%) Segmentation (61.4%) Motion (49.1%) Unmasking (15.8%) Blink (8.8%) Doubling of the retinal vessels (1.7%) Out of window (1.7%) Masking (1.7%) Projection (0.0%) Stretch artifacts (0.0%) Crisscross (0.0%)	Topcon OCT instrument (DRI OCT Triton plus, Topcon, Tokyo, Japan).
Al-Sheikh et al, 2017^[23]^	Prospective comparative study	Healthy subjects	Band (17.64–70.58%) Segmentation (5.8–11.6%) Motion (5.8%) Projection (0–47.05%)	DRI OCT Triton, TOPCON Inc., Tokyo, Japan
Say et al, 2017^[20]^	Observational study	Treated unilateral posterior uveal melanoma and fellow eye	Loss of focus (55%) Broad blink lines (37%) Motion (26%) Specular dot (25%) Edge duplication (8%)	The Optovue RTVue XR AVANTI, version 2014.2.0.13
Holmen et al, 2019^[15]^	Cross-sectional study	Diabetic retinopathy	Eye movement (93.1%) Defocus (74.9%) Shadow (62.3%) Tilt (50.5%) Z offset (43.8%) Refraction shift (31.8%) Segmentation (24.6%) Decentration (21.4%) Projection (6.7%) Blink (1%>) Stretch artifact (1%>) Edge duplication (1%>) Loss of signal (1%>)	CIRRUS HD-OCT 5000, with the AngioPlex module, version 10.0.0.13424 or the Optovue Avanti RTVue XR, version 2018.0.0.5).
Enders et al, 2019^[21]^	Prospective observational study	Healthy subjects Neovascular age-related macular degeneration Diabetic retinopathy Retinal vascular occlusions	Projection (100%) Segmentation (55%) Motion (49%) Masking (45%) Band (43%) Blink (28%) Window artifact (8%) Vessel doubling (0.0%) Stretch artifacts (0.0%)	Spectral domain OCT Cirrus 5000 equipped with the AngioPlex module)
Iftikhar et al, 2019^[16]^	Prospective cross-sectional study	Multiple Sclerosis Healthy subjects	Motion (96.3%) Blink (51.9%) Loss of focus (25.1%)	SpectralisⓇ OCT-A prototype, OCT2 (Heidelberg, Germany)
Eastline et al, 2019^[68]^	Prospective observational study	Healthy eyes	Displacement artefact (96.34%) Shadowing (92.27%) White line artefacts (63.41%) Vessel doubling (35.37%).	PLEX Elite 9000 scanner (Carl Zeiss Meditec, Inc., Dublin, CA). central 3 * 3 and 12 * 12 mm scans of the four quadrants (wide field)
Weijing et al, 2020^[17]^	Retrospective observational study	Glaucomatous eyes Healthy eyes	Projection (100%) Motion (75.22%) Blink (2.62%) Stretching (0.87%) Blurred images (24.78%) Decentration (21.28%) Vignetting (2.04%) Unmasking (0.87%) Segmentation (1.17%) Out of window (0.29%) Vessel doubling (0.58%)	N/A
Bontzos et al, 2020^[69]^	Prospective observational study	Eyes with idiopathic Epiretinal membranes Healthy eyes	Segmentation (0% in healthy eyes, 64.1% in ERM patients) Motion (7.5 % in healthy eyes, 53.8 % in ERM patients)	AngioVue, RTVue XR Avanti SD-OCT, Optovue Software version: 2016.2.0.35

**Table 2:** Different types of artifacts in optical coherence tomography angiography images.


**Type of artifact**		Definition
Motion	Blink	End-to-end black band
		Displacement	Waviness or discontinuity of the retinal vessels
		Doubling	Duplication of vessels
		Stretch	Stretched vessels or presence of linear bands at the edge of OCTA image (edge duplication)
		Quilting (Crisscross)	Rectangular or checker-board pattern
Band		Bands with various brightness
Segmentation		Retinal boundaries Misidentification
Projection		Presence of false flow in the avascular area
Projection removal		Traces left in the deeper layer after the removal of projected superficial vessels
Masking		Light blockage
Unmasking		Light hyper-transmission
Shadow		Ghost image of the superficial retinal vessels on the deeper layer
Z offset (out of window)		Vertical misaligned B-scans on the screen
Tilt		More than 50% of B scans are not focused clearly
Refraction shift		differing reflectivity of adjacent B-scans (probably the same as banding artifact)
Decentration		Not well-centered on the macula
Defocus		Whole B cans are not focused well
Suspended scattering particles in motion (SSPiM)		extra-vascular OCTA signals corresponding to hyperreflective intraretinal fluid
Fringe washout		Dark appearance of choroidal vessels

Holmen et al^[15]^ reported at least one artifact in 97.3% of images. Severe artifacts were recognized in 53.5% of scans and the three most common artifacts were shadow (26.9%), defocus (20.9%), and movement (16%). Artifact prevalence did not differ among imaging systems or scan protocols.

In a more recent study,^[17]^ 88.34% of OCTA images of the superficial vascular plexus of 343 eyes of 183 subjects including 100 glaucoma patients and 83 healthy participants showed at least one type of artifact. The most common artifact was projection (100%) followed by motion artifact (75.22%). Stepien et al^[14]^ reported that vessel density (VD) in 74% of eyes with retinal disease and 54.7% of normal subjects was unreliable due to artifacts. In 72% of images with unreliable VD, more than one artifact was found. Say et al^[20]^ reported a higher frequency of artifacts in eyes with underlying pathology or low vision and described loss of focus followed by broad blink lines (55 and 37%, respectively) as the most common artifacts in eyes with unilateral choroidal melanoma.

In another study, projection artifacts, segmentation errors, and motion artifacts were reported in 100%, 55%, and 49% of eyes of 6 healthy eyes and 69 eyes with underlying retinal disorders (including AMD, DR, RVO, and retinal artery occlusion), respectively.^[21]^ Iftikhar et al^[16]^ reported that some degree of artifact was noticed in 97.1% of images from healthy subjects and patients with multiple sclerosis. The most frequent artifact was motion artifact (96.3%). The probability of motion artifacts in these patients was higher in those with longer disease duration or history of optic neuritis. Ghasemi Falavarjani et al^[22]^ reported that 33% of healthy eyes and 100% of diabetic eyes showed segmentation errors.

All studies reported a higher prevalence of artifact(s) in eyes with underlying pathologies. In addition, low image quality has been reported to be associated with a higher prevalence of artifacts.^[15,23]^

Different OCTA devices utilize various propriety algorithms to detect, process, and visualize decorrelation signals. Relatively rapid advances in software updates to reduce the artifacts impede practical comparison of these devices. In a study by Li and colleagues^[24]^ evaluating the clinical performance of five OCTA devices (AngioVue, Angioplex, Spectralis, Angioscan, and OCTA SS OCT Angio${}^{\mathrm{TM}}$), the authors found that AngioVue had the least motion artifacts.

Theoretically, swept source (SS) OCTA instruments employing longer wavelength light are less affected by mask artifacts as compared to spectral domain OCTAs. Reich et al showed that SS-OCTA can mitigate shadow artifacts imposed by subretinal fluid on the choriocapillaris in the subjects with acute central serous chorioretinopathy and retinal detachment.^[25]^

Studies investigating artifacts in disc OCTA are scarce. The frequency of various artifacts and predisposing factors in disc OCTA have yet to be determined. In a study by Moghimi and colleagues,^[26]^ 20% of disc OCTA scans were graded as poor quality images. Similarly, Rao and colleagues^[27]^ found that 17% of disc OCTA scans had poor quality precluding useful interpretation.

### Types of Artifacts 

Table 2 provides an overview of artifacts. Artifacts can be categorized as patient-related (e.g., motion), software-related (e.g., motion, stretch, etc.), and operator-related (e.g., defocus). Some artifacts can be related to more than one subset of these categories (e.g., motion artifact).^[15]^

#### Artifacts associated with eye movement 

Any eye movement can lead to image artifacts. The cardiac cycle, breathing, tremors, and microsaccades cause pulsations. The consequence of these pulsations is motion of the choroid and retina.^[9,19]^ Several types of artifacts associated with eye movement including motion artifacts, doubling of retinal vessels, blink artifacts, stretch artifacts, and crisscross artifacts have been reported.^[9]^

Lauermann et al^[28,29]^ categorized motion artifacts under two groups: those caused by eye movement (blink lines and displacement) and artifacts due to software correction of eye movement (stretch artifacts, quilting, and vessel doubling).

Blink lines are caused by eye closure during image acquisition and result in loss of information. Lost adjacent B-scans cause end-to-end black bands with a width dependent on the duration of eye closure.^[13,29]^ Displacement of multiple adjacent B-scans leads to linear distortion of an image seen as waviness or discontinuity of retinal vessels.^[13,30]^

**Figure 1:**
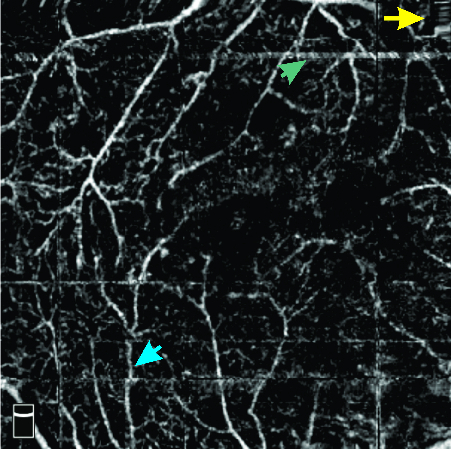
Crisscross artifact as well as stretching artifact (yellow arrow), white line artifact (green arrow) and displacement of the course of the retinal vessel (blue arrow).

**Figure 2:**
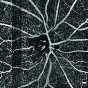
Banding artifact. Note a band with different brightness.

Doubling artifacts are defined as duplication of vessels or appearance of two or more similar non-overlapping images caused by software correction of eye motion.^[13,29][30]^ Stretch artifact is the result of motion artifact correction by the machine software. Intermittent changes in signals causes edge duplication which presents as a linear streak at the edge of an en-face image.^[16,29]^ Furthermore, stretched vessels appear to be flattened.^[13]^ Quilting (crisscross or checkerboard defect) is a result of failure of the software to correct multiple saccades. Quilting appears as a rectangular pattern of artifacts [Figure 1].^[29]^ While Lauermann et al considered quilting artifact as banding or checkerboard,^[29]^ Ghasemi Falavarjani et al^[13]^ provided different definitions for banding artifact, in which multiple adjacent B-scans form bands of different brightness compared to neighboring areas on en-face OCT or OCTA images. Banding artifact [Figure 2] is thought to be caused by a temporary change in corneal refractive power during blinking which causes a part of the image to be out of focus (also known as refractive shift).^[15]^ Introduction of software with fast and accurate tracking has significantly reduced the rate of motion-related artifacts.^[13]^

**Figure 3:**
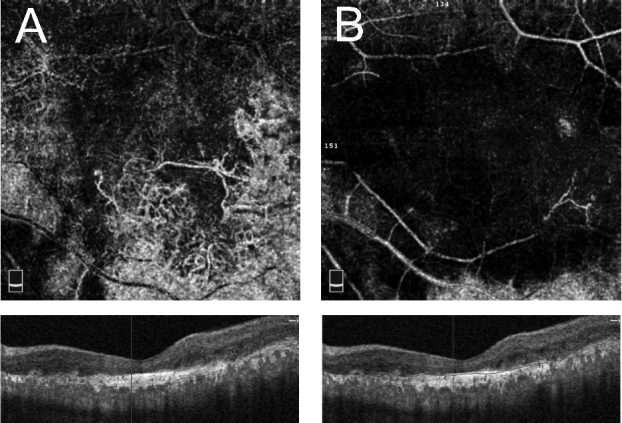
Segmentation error artifact. (A) en face optical coherence tomography angiography (OCTA) at segmented outer retinal slab with segmentation error in delineation of retinal pigment epithelium, showing large choroidal vessels masquerading as neovascularization. (B) en face OCTA at outer retinal slab after segmentation correction. Note that large choroidal vessels are now eliminated.

**Figure 4:**
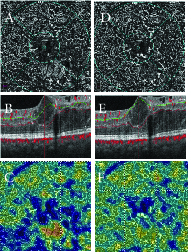
Enface OCTA, structural OCT and vessel density map of deep capillary plexus in a subject with diabetic macular edema, before (A–C) and after (D–F) segmentation correction at inner plexiform layer (green line) and outer plexiform layer (red line). Note a significant change in Enface OCTA and vessel density map following segmentation correction.

**Figure 5:**
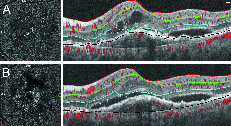
Misidentification of Bruch`s membrane (Black line) in a subject with macular neovascularization (A), following manual correction of Bruch`s membrane (B). Note better delineation of neovascular network after segmentation correction.

**Figure 6:**
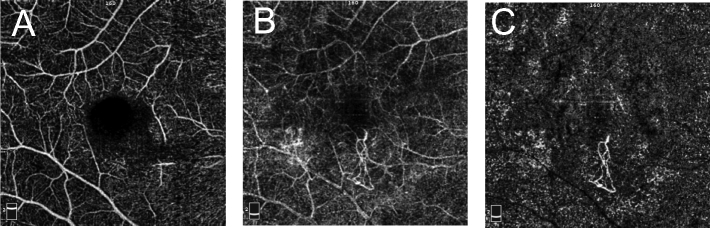
Projection Artifact. (A) en face optical coherence tomography angiography (OCTA) at superficial capillary plexus. (B) en face OCTA at outer retinal slab. Note prominent projection of superficial vessels, making detection of type 2 macular neovascularization challenging. (C) en face OCTA of outer retina after projection artifact removal using Angiovue software. Note the outline of neovascular tuft is now clearly visible and projection of the superficial capillary plexus removed.

**Figure 7:**
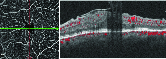
Masking (shadow) artifact in enface optical coherence tomography angiography and optical coherence tomography B scan in a subject with vitreous opacity.

**Figure 8:**
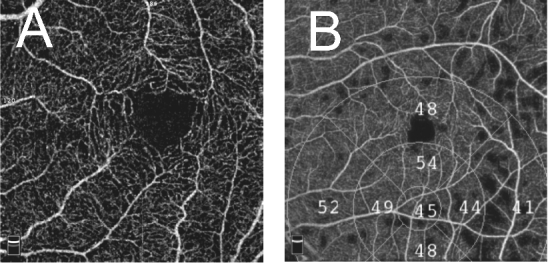
Decentration artifact. (A) Decentration of fovea in en face optical coherence tomography angiography (OCTA) at the level of superficial capillary plexus. (B) Decentration of the grid over en face OCTA vessel density map.

#### Misidentification of retinal layers (segmentation artifact) 

OCTA en-face images show the microvascular network in different slabs. Slabs are tissue layers limited by two retinal layer boundaries and are commonly divided into the superficial plexus, the deep plexus, the outer avascular retina, and the choriocapillaris.^[28]^ Any error in detecting the correct position of retinal boundaries leads to segmentation artifacts [Figures 3–5].^[13]^ A recent study defined segmentation error as a deviation exceeding 50% of the thickness of the pertinent plexus.^[15]^ Segmentation errors are more prevalent in low-quality images and in eyes with retinal pathologies.^[21]^

#### Projection artifact

OCTA imaging is based on detection of a significant change in light characteristics (intensity, phase, or a combination), reflected from the same location at short intervals. These changes are then attributed to moving blood cells within the vessels. However, the transmitted light through a vessel may be erroneously perceived as flow when it is reflected from underlying reflecting surfaces (e.g. RPE).^[9]^ Therefore, projection or tailing artifacts are the presence of false flow in deeper slabs [Figure 6].^[31]^ Projection artifact should be considered if vessels in the deep capillary plexus (DCP) appear to have the same pattern as the superficial vessels.^[10]^ In addition, projection of superficial retinal vessels on deeper layers^[9]^ should be considered in the evaluation of choroidal neovascularization, because projected images from either the superficial retinal vessels or intraretinal migrant pigments may be misinterpreted as CNV.^[9]^ This is particularly important in cases with retinal pigment epithelium detachment (RPED), because in these cases, the highly reflective nature of the RPE induces a projection artifact that appears as a bright ring at the edge of a PED.^[32]^ Chen et al demonstrated that RPE hyperplasia overlying PED may cause false flow signal in deeper layers.^[33]^

Projection artifact can be used to examine the anatomy of the choroidal vessels. By moving and placing the segmentation line behind the choroid, the vascular pattern of choroidal vessels can be projected onto the sclera.^[34]^

Although recently updated software programs can efficiently remove projection artifacts, another artifact may be introduced by eliminating the projection artifact. This “projection removal" artifact is defined as obscuration of vessels in deeper layers due to removal of projected superficial vessels by the device software.^[10]^ The details of projection artifact removal (PAR) algorithms is beyond the scope of this review. The main idea is taking account of the OCT and OCTA parameters of a given point in relation to its neighboring and anterior structures to differentiate true flow from the projections.^[35]^ Despite advances in PAR algorithms, Fayed and Fawzi^[35]^ demonstrated that commercial PAR-OCTA might not be able to completely eliminate false flow associated with hard exudates and pigment migrations in retinal angiomatous proliferation.

#### Low-OCT-signal artifacts

Numerous factors may contribute to low OCT signals including vignetting, ocular aberrations, system aberrations, angle-dependent backscattering, retina moving out of focus, signal roll-off, any media opacity, intra- or sub-retinal fluid and hemorrhage, vascular shadowing, and RPE clumping. Vignetting is one of the main causes of low-OCT-signal artifacts and is exacerbated with increased field size and smaller pupil diameter. Vignetting may occur as a result of partial or complete blockage of the incident beam by the iris.^[36]^ Low OCT signals may result in segmentation error, as described above.

De Pretto et al proposed three strategies to detect low OCT signal on OCTA images.^[36]^ In the simplest and most reliable approach, namely, cross-sectional approach, careful B-scan-by-B-scan analysis, or B scan fly-through is performed to detect areas of low signal or segmentation errors. In the en face approach, OCT and OCTA en face images are compared alongside. Low-OCT-signal leading to segmentation error creates an abrupt signal alteration on en face OCT images. The en face approach provides a general view of data and facilitates the recognition of artifacts around the lesion of interest. In orthoplane approach, which is a combination of the two former strategies, an en face approach is used to recognize areas of low OCT signal and then a cross-sectional analysis is employed to assess these areas.^[36]^

OCTA thresholding is the procedure for removal of areas with low or noisy OCT signals. If OCTA thresholding does not take place, low OCT signal areas form regions with low OCTA signal, independent of presence or absence of blood flow. This is named thresholding artifact.^[36,37]^

Light blockage as a result of more anterior lesions including vitreous opacities, pigment clumps, etc. does not permit the beam to reach deeper layers. This causes masking or shadow artifacts [Figure 7].^[13]^ On the contrary, the excess transmission of light due to RPE or retinal atrophy gives rise to increased OCT reflectivity which is labeled as unmasking or hyper-transmission.^[10,13]^

Although OCTA has been shown to be superior to conventional dye-based angiography in detecting macular neovascularization in the context of macular atrophy,^[38]^ special emphasis should be laid on the interpretation of findings. Anterior displacement of larger choroidal vessels along with unmasking artifact in areas of geographic atrophy may falsely create the impression of macular neovascularization.^[39]^

“Ghost images" are shadows of large superficial retinal vessels on deeper layers that impede extraction of vascular information from the areas beneath these vessels.^[8]^ In other words, masking artifacts from large retinal vessels are considered as shadow artifacts.^[19]^ Shadow artifacts occasionally refer to the attenuated signals caused by an opacity or an obstruction due to retinal bleeding, subretinal fluid, or drusen.^[25]^

#### Wide-field OCTA

Wide-field OCTA provides a wider field of view compared to the traditional 3x3 and 6x6 images. There are several factors that may contribute to increased prevalence of image artifacts in wide-field OCTA. Longer image acquisition time may lead to poor patient cooperation and increased motion artifacts. Peripheral regions might get out of focus due to retinal curvature. The longer wavelength of SS-OCTA has lower axial resolution which may result in lower contrast between retinal layers and consequently higher rates of segmentation errors. A wider field of view is more sensitive to OCT beam–pupil misalignment and low-signal artifacts.^[36,40]^ In addition, there are some artifacts specific to wide-field imaging. Alignment error is unique to montage OCTA images and is understood to be caused by projection removal in processing of depth-coded images. In this artifact, the superior and inferior half of the image appears to origin from different depths.^[40]^ Eyelash artifact has recently been described on wide-field images as a new subset of shadow artifact.^[41]^

#### Other artifacts 

Some artifacts are less frequent. Z-offset (or out of window) artifact results from vertical displacement of B-scans on the screen.^[13,15]^ Tilt artifact occurs in the presence of severe angle of incidence, head placement, and/or high myopia which result in half of the B scans being defocused.^[15]^ A refraction shift artifact can occur with an alteration in reflective intensity between adjacent scans due to blinking or an alteration of corneal surface refractive index. Refraction shift artifact is ostensibly a subtype of banding artifact as described before.^[15]^

Decentration artifact arises when a scan is not centered on the macula [Figure 8].^[15]^ In addition, the ETDRS grid that is overlaid on the VD map, may fail to detect the center of the fovea, previously described as grid decentration artifact on structural OCT images.^[42]^

Defocus artifact is defined as reduced definition of retinal microvasculature on en face OCTA images and decreased reflectivity of inner retinal layers compared to normal standard OCT images and is caused by a defocused image.^[15]^

Suspended scattering particles in motion (SSPiM) are responsible for nonvascular decorrelation signals in hyperreflective fluid associated with various exudative maculopathies including DR, RVO,^[43]^ and Coats disease.^[10]^ Brownian movements of lipoproteinacious particles in intraretinal cysts, similar to moving red blood cells (RBC) in vessels, are detected by OCTA instruments. These signals are not representative of RBCs within vessels and may be considered as artifacts. Maltsev et al^[44]^ demonstrated that the presence of SSPiM in eyes with diabetic macular edema may artefactually increase vessel densities in the DCP when a 3-mm scan protocol is employed.

Hyperreflective crystalline deposits in AMD can produce multiple hyper-intense vertical lines passing through these lesions, extending anterior and posteriorly in cross-sectional B-scans.^[45]^

Fringe washout artifact occurs in en face choroidal slabs. In contrast to retinal vasculature, choroidal vessels appear as cord-like dark vessels. This poor backscattered signal is in contrast to the surrounding hyper-reflective choroidal stroma and allows visualization of the vessel outline. Fringe washout artifact occurs in Sattler's and Haller's layers.^[19,46]^ To point out the nature of the hyper-reflective area around dark choroidal vessels, Maruko et al^[47]^ proposed that the surrounding whitish area is due to projection artifact from the overlying choriocapillaris layer. While an hourglass signal pattern is present in large retinal vessels, choroidal vessel lumen seems dark. If RPE atrophy happens, an hourglass pattern in choroidal vessels appears. Therefore, it seems that the “masking” effect of RPE is the main cause for choroidal vessels appearing as dark regions.^[48]^

Movements in the vessels and consequently, flow signal can be detected even if the laser beam is not centered on the vessel.^[9]^ Therefore, vessel diameter may be erroneously displayed. Ghasemi Falavarjani et al showed that in OCTA images, vessel diameter measurements were significantly larger than those obtained on color fundus photographs, particularly for smaller vessels.^[49]^

### Artifacts Grading System

Some articles suggest a scoring system for artifact grading. However, the proposed systems are not widely employed. A simple grading protocol was introduced by Munk et al^[50]^ to compare four OCTA devices. They graded artifacts as motion artifacts (1 = no artifacts, 0 = some artifacts, –1 = severe motion artifacts) and image artifacts (1 = no artifacts, 0 = some artifacts, –1 = severe image artifacts). The image artifact category included segmentation and projection artifacts.

For grading motion artifacts, a motion artifact score (MAS; scores I–IV) has been proposed.^[28,29]^ The grading ranges from MAS 1 (no or slight quilting, absence of other motion artifacts) to MAS 4 (artifacts in more than two quadrants, with either moderate or significant quilting, displacement, vessel doubling, stretch artifact).

For evaluation of segmentation accuracy, Lauermann et al^[28]^ introduced the segmentation accuracy score (SAS; score I–IIB) that can be used in all retinal diseases. More than 50 μm deviation of segmentation from the correct reference plane was defined as inaccurate. Presence of inaccurate segmentation in <5% of all single B scans; in each boundary (ILM, IPL, or CC) is graded as SAS I. If this happens in >5% of scans, it is defined as SAS II. The involvement of only one reference plane is categorized as SAS IIA, while the presence of errors in more than one plane is defined as SAS IIB.^[28]^

Later a more general grading system was introduced by Holmen et al.^[15]^ They described a severity scale of 0 to 3 for each artifact, in which 0 is no artifact and 3 is the appearance of artifacts in >10% of OCT B-scans within the inner or central subfield.

##  DISCUSSION

The ability of OCTA to capture microvascular network images in different retinal layers and its high resolution and high speed characteristics renders it as a promising and invaluable imaging modality for the diagnosis and management of posterior segment diseases.^[51]^ However, the impact of artifacts on the interpretation and precision of OCTA-derived metrics should be cautiously monitored.^[15]^ Artifacts are frequently observed on OCTA images and may occur during image acquisition, processing, and analysis.^[52]^ Low image quality and underlying posterior segment pathology are associated with a higher prevalence of artifacts. The frequency and types of artifacts may differ according to the underlying disease. Furthermore, a lesion may cause more than one type of artifact.

The frequency and severity of artifacts may be influenced by the type of OCTA device. Some artifacts such as segmentation artifacts and duplication of vessels have been reported to be more dependent on the type of OCTA device; however, motion artifacts, either in SCP or DCP images, are less dependent on the type of OCTA machine ^[24]^. The frequency of different types of artifacts varies among layers. While motion and banding artifacts are common in superficial and deep retinal layers, segmentation and projection artifacts are more prevalent in deep retinal layers. Masking artifacts occur more frequently in the choroidal layer.^[21]^

With growing popularity of wide-field imaging in clinical practice, artifacts in wide-field OCTA images warrant special attention. Failure to recognize and address artifacts on wide-field images can lead to incorrect diagnosis of peripheral non-perfusion or inability to visualize retinal neovascularization. This issue is especially important in evaluation of non-perfusion areas (NPA) in DR because low signal artifacts can masquerade as NPA.^[36,41]^

In a study by Pichi et al, manual segmentations was necessary in the majority of eyes to enhance the characterization of neovascularization.^[53]^

Some general practical measures may be employed to minimize artifacts in OCTA imaging. Proper attention should be paid to identify the subjects more prone to artifacts. These include systemic conditions such as Parkinsonism or ocular pathologies including retinochoroidal diseases or ocular surface disorders. Ocular surface conditions should be optimized in eyes with dry eye disease by instilling artificial tears. Dilating eye drops should be used for wide-field imaging. Instructions should be provided for patients regarding the procedure. Stable and comfortable position and regular breaks during image acquisition are crucial. Proper transverse and axial alignment of the OCT beam may be judged by checking the retinal B scans.^[36]^

Strategies to reduce motion artifacts can be applied during or after image acquisition.

Eye-tracking systems and scanning protocols can help curtail artifacts during image acquisition, and different algorithms can be utilized to reduce motion artifacts after taking the images.^[54,55]^ Although post-processing motion correction techniques may not tolerate gross saccadic motion and induce additional artifacts, a combination of post-processing algorithms along with modified scanning protocols and eye-tracking systems are promising.^[54,55]^

Automated segmentation algorithms may cause incorrect recognition of layers,^[22]^ especially in the peripapillary area.^[56]^ This misalignment can be corrected by the “Edit Band/Propagation” tool on the Optovue device software. By using this tool, users can fix one or few B-scans and propagate the correction to the rest of the adjacent scans.^[22,56,57]^ Ghasemi Falavarjani et al^[58]^ demonstrated that by implementing stepwise correction of segmentation lines, it is possible to reach complete correction by addressing this error on a relatively few number of B-scans in eyes with diabetic macular edema. Notably, correction of central foveal B-scan had the most significant impact on VD measurements. Recently, Hanna and colleagues^[56]^ showed that automated peripapillary retinal segmentation using the Spectralis device may lead to underestimation of vessel densities at nerve fiber layer vascular plexus in normal and glaucomatous eyes. The process of manual segmentation, however, was time-consuming (5 hr per eye) and thus not practical in clinical settings. Correcting segmentation error artifacts is more important when comparing OCTA metrics in longitudinal studies.^[59]^

In the short term, manual correction of segmentation lines appears to be the best practical method to tackle segmentation errors. However, recent advances in deep learning/machine learning might be superior using to fixed models for retinal segmentations. Various techniques of machine learning including support vector machines^[60]^ and neural networks^[61,62]^ have been employed and appear to be promising.

Different strategies have been implemented to address projection artifacts. These include simple superficial vessel subtraction and different projection resolved (PR) algorithms.^[35,63,64]^ It has been shown that PAR software can alter SCP and DCP VD measurements and may interfere with VD assessment. This should be considered when comparing studies reporting VD using different software updates.^[12,65]^

Evaluation of choriocapillaris blood flow in dry AMD demonstrated changes in vascular density of the choriocapillaris.^[66]^ Confounding factors including projection and shadow artifacts can affect estimation of non-perfusion areas in the choriocapillaris. Shadows on the choriocapillaris may originate either from large retinal vessels or overlying drusen. In case of any suspicion about flow impairment in the choriocapillaris in patients with macular lesions, flow images should be interpreted alongside structural en face images and OCT B scans.^[67]^ SRF can affect the choriocapillaris OCTA signal. Swept-source OCT technology utilizes a longer wavelength and higher scan speed, therefore shadow artifacts are reduced with this generation of machines.^[25]^

In summary, image artifact is a major concern in the interpretation and quantification of OCTA images. Despite recent advances in OCTA technology, there is an emerging need for eliminating image artifacts. Retinal specialists and OCTA technicians should be familiar with different types of artifacts and strive to eliminate or minimize them. The most important aspect is allocating enough time and attention for detecting possible artifacts on OCTA images. Adding structural OCT image data and 3D evaluation of the images are crucial. There is an emerging need for developing a grading system for artifacts. However, given their qualitative nature, consensus on a universally accepted grading system poses certain challenges. Remote OCTA-viewing software may help clinicians to look for the artifacts in a systematic approach.

##  Financial Support and Sponsorship

Nil.

##  Conflicts of Interest

There are no conflicts of interest.
